# Leaf- and canopy-level chlorotic symptom variations are associated with differential *Candidatus* Liberibacter asiaticus infection patterns in Ponkan mandarin under field conditions

**DOI:** 10.1128/spectrum.00760-26

**Published:** 2026-07-02

**Authors:** Yu-Min Wu, Chia-Ching Chu

**Affiliations:** 1 Department of Plant Pathology, National Chung Hsing University 34916, Taichung, Taiwan; USDA-ARS San Joaquin Valley Agricultural Sciences Center, Parlier, California, USA

**Keywords:** citrus greening, molecular detection, disease progression, fastidious bacteria

## Abstract

Ponkan mandarin (*Citrus reticulata* cv. Ponkan) is an important fruit crop planted across different countries. One of its most devastating diseases is citrus huanglongbing (HLB), which is associated with infection by the bacterium *Candidatus* Liberibacter asiaticus (*C*Las). Although studies on HLB in Ponkan mandarin have been conducted, symptom heterogeneity and its association with *C*Las titer under field conditions remain underexplored. We examined the association between symptom variation and *C*Las infection status in a Ponkan mandarin grove by categorizing symptoms at both the leaf and canopy (tree) level. Chlorotic leaves were sampled and classified into different categories, including leaves exhibiting interveinal chlorosis (Cat. A), those showing vein yellowing (Cat. B), and blotchy mottled leaves (Cat. C). Polymerase chain reaction (PCR) and quantitative PCR (qPCR) assays targeting *C*Las revealed that Cat. A leaves, which resembled nutrient-deficient leaves, had higher *C*Las detection rates and a larger proportion of samples with high *C*Las titers. Canopy-level assessment of symptom diversity within each tree also revealed that plants bearing Cat. A leaves were more likely to carry *C*Las and have high pathogen loads. The likelihood was also greater in trees with higher leaf symptom diversity. Additional prophage typing of the detected *C*Las strains revealed that leaf symptom variations were independent of prophage type difference. Nutrient analyses revealed reduced magnesium, manganese, and zinc contents in Cat. A leaves, indicating that leaves prone to *C*Las infection also exhibited nutrient deficiencies. These findings highlight the complexity of potential cultivar-specific associations between *C*Las and citrus under field conditions.

**IMPORTANCE:**

Although it is well-established that HLB in Ponkan mandarin is associated with *C*Las infection, the relationship between leaf symptom variation in this cultivar and the presence and titer of *C*Las under complex field conditions remains unclear. By examining symptom heterogeneity in natural field conditions at both the leaf and canopy level, this study demonstrated that HLB leaf symptom variation in naturally infected Ponkan mandarin corresponds to differences in *C*Las presence and titer at both levels. Notably, although blotchy mottling is considered the typical leaf symptom of HLB, we found that *C*Las detection rates and titers tended to be greater in leaves showing nutrient deficiency-like symptoms, which contrasts with patterns in other citrus cultivars. Recognizing inconsistencies among cultivar-specific patterns is important for interpreting HLB symptoms, devising sampling strategies, and exploring uncharacterized factors affecting symptom expression under field conditions.

## INTRODUCTION

Citrus are important fruit crops cultivated across the world (1). Selective breeding and cross-hybridization have resulted in highly diverse cultivars that vary in morphology and physiology (1, 2). Like many other commercial crops, citrus are subject to diseases caused by various types of microorganisms and parasites, including bacteria (3).

Citrus huanglongbing (HLB) or citrus greening is one of the most important diseases of citrus (4–6). The occurrence of HLB in Florida since 2005 has been associated with a decline in citrus production (7) and has led to billions of dollars in annual economic loss (8). In countries like China and Brazil, HLB also led to the removal of significant numbers of citrus trees (6, 9, 10). The disease is associated with infection by three species of the bacterial genus *Candidatus* Liberibacter, namely *Ca*. Liberibacter asiaticus (*C*Las), *Ca*. Liberibacter africanus (*C*Laf), and *Ca*. Liberibacter americanus (*C*Lam). These Gram-negative, phloem-inhabiting bacteria can be transmitted by sap-sucking insects of the superfamily Psylloidea (i.e., psyllids) or by grafting and transportation of infected plant materials (4, 11–13). After invading the phloem, liberibacters can induce diverse symptoms in different plant parts, such as leaf chlorosis, corky veins, defoliation, reduction of fruit size, lopsided fruit, and even death of the entire plant (4, 14–17). Among these symptoms, leaf chlorosis, more specifically the “blotchy mottling” symptom, has been considered one of the most typical indicators of HLB (4).

Of the three liberibacter species, *C*Las, primarily transmitted by the Asian citrus psyllid (*Diaphorina citri*), is considered the most widespread and economically important pathogen (4, 13, 18–22). Previous studies on genome and 16S rRNA gene sequences of different *C*Las strains have shown that despite their wide distribution, *C*Las strains are genetically uniform (23, 24). However, these bacteria often harbor one or more types of prophages and can be divided into different “prophage typing groups” (PTGs) based on the number and combinations of prophages present (23, 25–27). Initially, studies of *C*Las genomes have identified three double-stranded DNA prophages, SC1 (Type 1), SC2 (Type 2) (28), and P-JXGC-3 (Type 3) (29). More recently, a single-stranded DNA prophage, CLasMV1 (Type 4), was also identified (30). Interestingly, a study showed that a *C*Las strain carrying Type 1 prophage induced different leaf symptoms on the same citrus cultivar than a strain with a Type 2 prophage (31), suggesting that prophage type difference can have an effect on *C*Las-citrus interaction.

Although the association between *C*Las and HLB is well-established (4, 13), leaf symptom expression in citrus does not always correlate well with *C*Las presence and titer, and may result in diagnostic uncertainty, epidemiological misinterpretation, or limitation in causal inference (32–34). Uneven distribution and low-titer infection of *C*Las (33, 34) can confound data interpretation. Citrus cultivar differences and *C*Las strain variations may lead to different HLB symptom development (15, 35, 36). Leaf symptoms observed within the same cultivar can also be very diverse (17). Furthermore, factors unrelated to *C*Las, such as nutrient deficiency, can induce leaf chlorotic symptoms resembling those of HLB (36–39). In this regard, field-based, cultivar-specific studies pairing standardized symptom phenotyping with *C*Las infection status can help to understand relationships between leaf symptom expression and *C*Las infection patterns on a cultivar-by-cultivar basis.

Ponkan mandarin is one of the primary loose-skin mandarin cultivars produced in Asia (40–42) and is commonly grown in Taiwan (43). Ponkan mandarin is also susceptible to HLB (36), and substantial research on *C*Las infection in this cultivar has been conducted. Topics from past studies have ranged from characterizing *C*Las strains infecting Ponkan mandarin, comparing the cultivar’s HLB susceptibility to other commercial cultivars, to examining gene expression or metabolite profile changes in plants following HLB infection (36, 40, 43, 44). However, to date, detailed understanding of how the presence and abundance of *C*Las correlate to Ponkan mandarin’s leaf symptom expression under field conditions remains lacking. In July 2021, a Ponkan mandarin grove in Miaoli County, Taiwan, was found to be affected by HLB after preliminary diagnostic PCR assays on leaf samples submitted by the growers tested positive for liberibacter. Because leaf symptom heterogeneity was observed in this specific area, the grove provided an ideal opportunity for investigating the association between *C*Las infection status and symptom variation. The present study categorized leaf chlorotic symptoms observed in the grove, and conducted polymerase chain reaction (PCR) and quantitative PCR (qPCR) assays to compare *C*Las infection statuses across different leaf symptom categories. Additionally, tree-level categorization and analyses were also conducted by considering the diversity of leaf symptoms found on each tree. We also examined whether different chlorotic symptoms were affected by the types of prophages found in *C*Las, and determined whether *C*Las infection and nutrient deficiency can co-occur in leaves with similar symptoms. The findings from this study provide implications for *C*Las ecology in Ponkan mandarin and may facilitate symptom interpretation and understanding of HLB progression trends under complex field conditions.

## RESULTS

### Confirming the presence and the identity of the liberibacter affecting the Ponkan mandarin grove

Initial PCR tests with liberibacter-specific primers LG774F and LG1463R (45) were first conducted on 65 samples collected from different trees in August 2021. Among them, 15 amplified a specific 684-bp amplicon (Fig. S1). Sequencing of amplicons in five representative samples revealed that they shared the same sequence; a representative sequence has been deposited in GenBank (accession no. PX363072). Examination of the chromatograms revealed well-resolved peaks in all positions, indicating absence of mixed infection. A BLASTN search against GenBank showed that the detected sequence was identical to the partial 16S rRNA gene sequences of many previously reported *C*Las strains, such as Ishi-1, gxpsy, A4, and psy62 (accession nos. AP014595, CP004005, CP010804, and CP001677, respectively; 639/639 bp). Comparing the obtained sequence against those of reference strains belonging to *C*Laf (accession no. EU921620), *C*Lam (accession no. CP006604), and another well-known pathogenic liberibacter, *Ca*. Liberibacter solanacearum (accession no. CP002371), revealed identities of only 97.5% (623/639 bp), 94.37% (603/639 bp), and 97.81% (625/639 bp), respectively.

### *C*Las detection rates in Ponkan mandarin leaves showing different symptom types determined by conventional PCR

**Fig 1:**
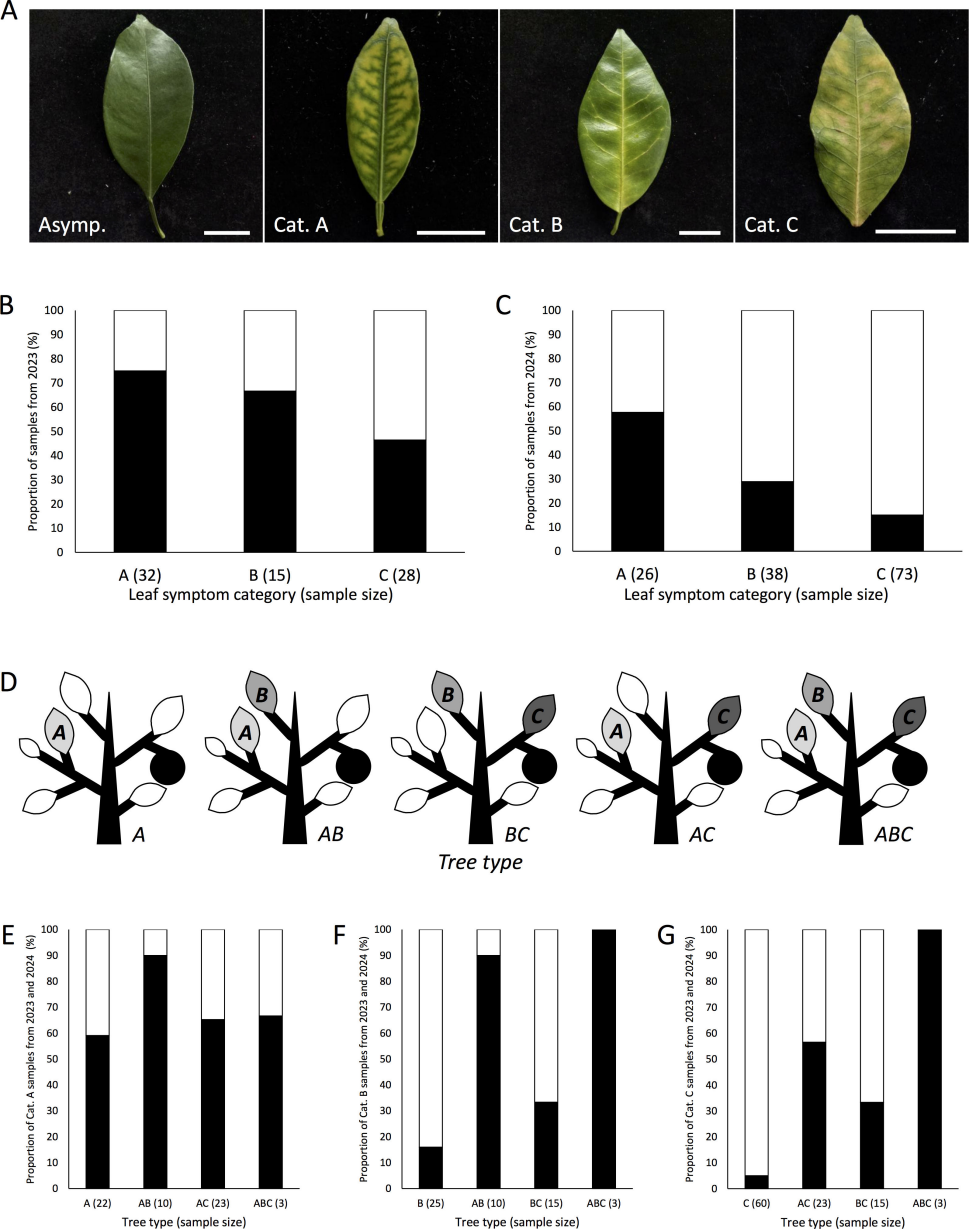
(**A**) Photos showing asymptomatic Ponkan mandarin leaves (Asymp.) and leaves belonging to three different symptom categories defined in this study (Cat. A, B, and C). Leaves in Cat. A exhibited interveinal chlorosis, those in Cat. B showed yellowing of veins, and leaves belonging to Cat. C exhibited blotchy mottling symptoms. The white scale bars indicate 2 cm. (**B and C**) *C*Las detection rates in symptomatic leaves of different categories collected in (**B**) August 2023 and (**C**) January 2024, determined with conventional PCR. The values are indicated with black bar segments. (**D**) Symptomatic trees sampled in this study were also grouped into different “tree types” based on the leaf symptom diversity found in each tree. (**E–G**) *C*Las detection rates of leaves belonging to (**E**) Cat. A, (**F**) Cat. B, and (**G**) Cat. C with “tree types” considered.

The association between leaf symptom variation and *C*Las detection rate was assessed with conventional PCR assays targeting *C*Las’s *omp* gene (Fig. S2A) and Ponkan mandarin’s resistance protein-like protein gene (Fig. S2B). Tests were conducted on larger sets of DNA samples extracted from symptomatic and asymptomatic (Asymp.) leaves collected in August 2023 and January 2024. Symptomatic leaves were grouped into different categories, including leaves exhibiting interveinal chlorosis (Cat. A; nutrient deficiency-like symptom), leaves showing yellowing of veins (Cat. B), and those exhibiting blotchy mottling symptoms (Cat. C; Fig. 1A). The asymptomatic leaves served as references. All samples were confirmed to be of sufficient quality, as indicated by successful amplification of the Ponkan mandarin gene.

In samples collected in 2023, the highest *C*Las detection rate was found in Cat. A samples (75.0%), followed by leaves grouped to Cat. B (66.7%) and Cat. C (46.4%; Fig. 1B). The difference among samples of the three symptom categories was marginally significant (*P* = 0.069; chi-square test). In leaves sampled in 2024, Cat. A samples exhibited a detection rate similar to that found in 2023 (Benjamini-Hochberg [BH]-adjusted *P* > 0.05; chi-square test), but Cat. B and Cat. C samples had lower detection rates compared with the previous year (BH-adjusted *P* < 0.05; chi-square tests). Nevertheless, the relative ranking of *C*Las detection rates among the three categories remained consistent across two time points. Statistical analysis revealed a significant difference among *C*Las detection rates of leaves belonging to different symptom categories (Cat. A to Cat. C; *P* = 0.00014; chi-square test; Fig. 1C) in 2024. The detection rate of Cat. A samples (57.7%) was significantly higher than those of the other two categories (28.9% and 15.1% for Cat. B and Cat. C, respectively; BH-adjusted *P* < 0.05; pairwise chi-square tests). All 88 and 45 asymptomatic samples, respectively, collected in August 2023 and January 2024, were tested *C*Las-negative.

To determine the correlation between *C*Las detection rates and symptoms at the canopy level, data analyses were also conducted while considering the symptom composition of the leaves in each tree. In such analyses, the plants were categorized into different “tree types”. For example, trees with only Cat. A leaves were classified as “Tree type A,” and trees with both Cat. B and Cat. C leaves were grouped into “Tree type BC” (Fig. 1D). Statistical analyses on pooled data from 2023 to 2024 showed that the *C*Las detection rates in Cat. A leaves from different tree types were not statistically different (*P* = 0.33; Fisher-Freeman-Halton test; Fig. 1E). Detection rates of both Cat. B and Cat. C leaves varied significantly across tree types (*P* < 0.0001; Fisher–Freeman–Halton test; Fig. 1F and G).

The *C*Las detection rate of Cat. B samples from “Tree type B” was significantly lower than those of “Tree type AB” and “Tree type ABC” (BH-adjusted *P* < 0.05; pairwise Fisher’s exact tests). Cat. B leaves from “Tree type AB” had a significantly higher detection rate than those from “Tree type BC” (BH-adjusted *P* < 0.05; pairwise Fisher’s exact tests). The *C*Las detection rate of samples from trees with Cat. C leaves alone was significantly lower than those of Cat. C leaves from the other tree types (BH-adjusted *P* < 0.05; pairwise Fisher’s exact tests). In both Cat. B (Fig. 1F) and Cat. C (Fig. 1G) samples, *C*Las detection rates were overall higher in tree types containing Cat. A leaves, although it should be noted that the sample sizes for trees containing all three leaf symptom categories were low (only three).

Among trees bearing leaves of only one category, those carrying Cat. A leaves alone had a higher *C*Las detection rate (59.1%) than those with Cat. B (16%) or Cat. C (5%) leaves (Fig. 1E through G).

### *C*Las titers in Ponkan mandarin leaves showing different symptom types in the grove-wide survey (determined by qPCR)

**Fig 2:**
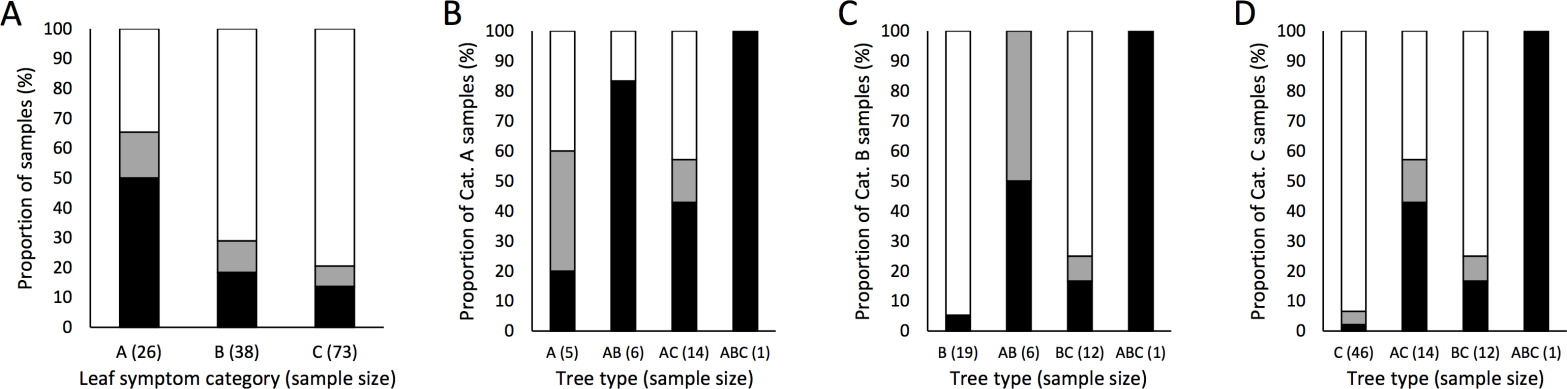
(**A**) Proportions of Ponkan mandarin leaf samples collected in January 2024 with different *C*Las infection statuses. The leaves were grouped into different symptom categories (Cat. A, B, and C). The black and gray segments indicate the proportions of samples with high (population densities ≥1) and low (population densities >0 and <1) *C*Las titers, respectively. The proportions of samples in which *C*Las was not detected (population density = 0) are indicated with white bar segments. Data of samples belonging to (**B**) Cat. A, (**C**) Cat. B, and (**D**) Cat. C with the “tree type” factor considered are also shown.

To study the association between leaf symptom difference and *C*Las titer, the population densities of *C*Las among samples from January 2024 were subsequently determined using qPCR. The *C*Las population density in each sample was represented as the copy number of a *C*Las gene per copy number of a Ponkan mandarin gene (see Materials and Methods section for details). *C*Las infection patterns were significantly different across symptom categories (*P* = 0.0008; Fisher-Freeman-Halton test; Fig. 2A). Cat. A leaves had the largest proportion of samples (50%) carrying *C*Las at high titers (population densities ≥1); adjusted residual analyses also showed that only the proportion value in Cat. A leaves was significantly higher than expected (BH-adjusted *P* < 0.05). Cat. B leaves had the second highest proportion (18.4%) of samples with high *C*Las titers, and the lowest proportion (13.7%) was found in leaves of Cat. C (Fig. 2A). Only 1 out of the 45 asymptomatic leaves serving as references was found to carry *C*Las at a low titer (population densities >0 and <1).

Although statistical analyses considering tree type differences for the qPCR data (from 2024) were not performed due to smaller subgroup sample sizes, the trends in the *C*Las infection rates determined were consistent with those observed in the conventional PCR assays (Fig. 1E through G). Additionally, among trees containing leaves of only one category, those bearing Cat. A leaves had higher proportions of samples with high *C*Las titers (Fig. 2B through D). When comparing the data of leaves belonging to Cat. B (Fig. 2C) and Cat. C (Fig. 2D), the proportions of samples with high-titer infection were overall greater in tree types containing Cat. A leaves. Again, such interpretation is limited to plants with one to two symptom categories, since samples from “Tree type ABC” were extremely scarce.

### *C*Las titers in leaves showing different symptom types within the same trees

**Fig 3:**
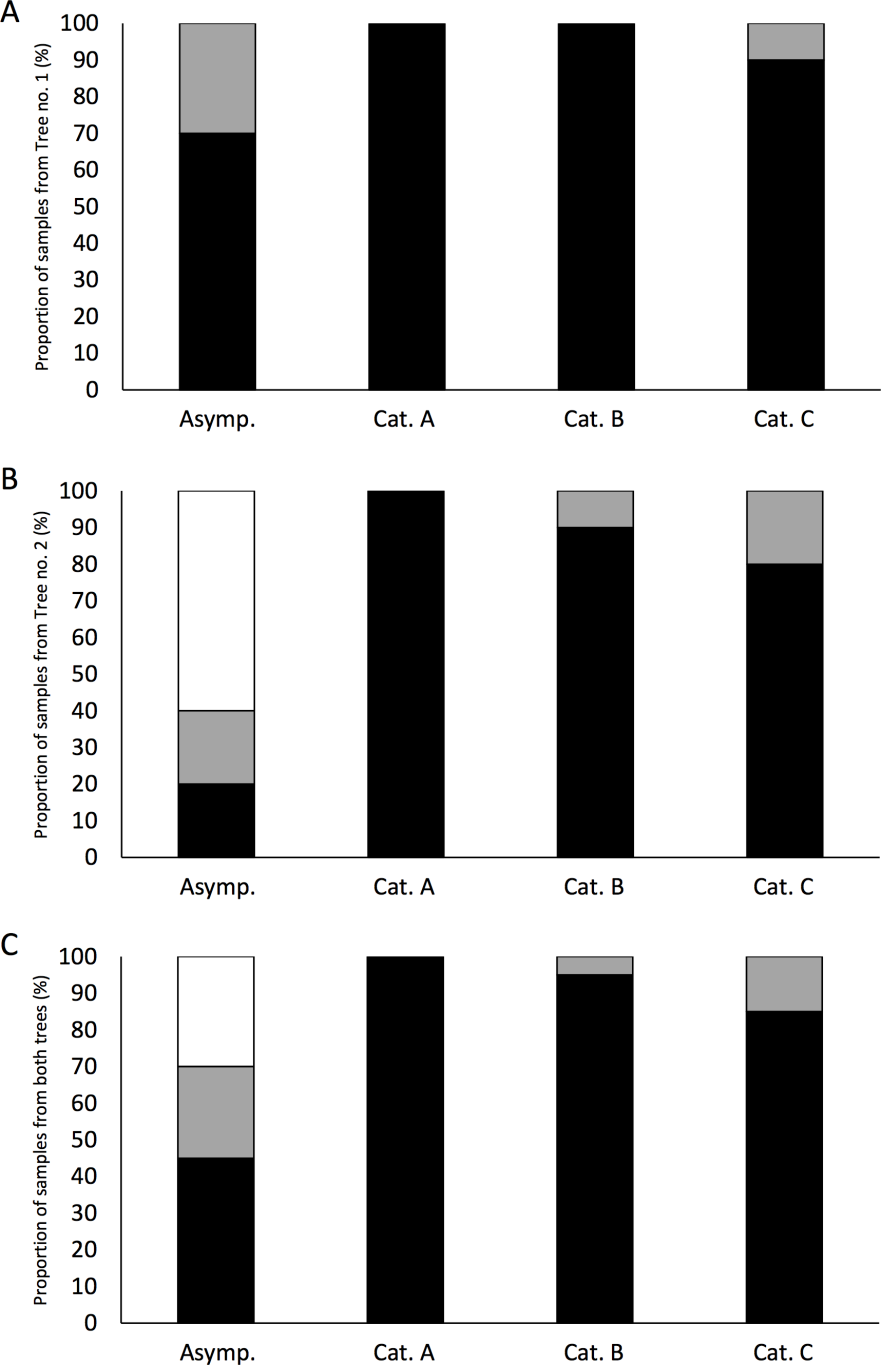
To control for tree-level variation, two sets of Ponkan mandarin leaf samples were collected from two trees, with each set including leaves that were either asymptomatic or exhibited symptom categories A, B, or C. (**A**) Data of samples from Tree no. 1. (**B**) Data of samples from Tree no. 2. (**C**) Combined data from the two trees. The black and gray segments indicate the proportions of samples with high (population densities ≥1) and low (population densities >0 and <1) *C*Las titers, respectively. The proportions of samples in which *C*Las was not detected (population density = 0) are indicated with white bar segments.

To control for tree-level variation, qPCR tests were also conducted on additional samples collected from two trees (Tree no. 1 and Tree no. 2) bearing leaves of all three symptom categories (as well as asymptomatic leaves). The data revealed that leaves exhibiting Cat. A symptom had larger proportions of samples carrying high *C*Las titers (100% in both trees; Fig. 3A and B). However, the proportional differences across leaf symptom categories were not prominent; 90%–100% and 80%–90% of Cat. B and Cat. C leaves, respectively, had high-titer infections. As high as 100% and 40% of the asymptomatic leaves collected from Tree no. 1 and Tree no. 2 were respectively tested *C*Las-positive, among which 70% and 20% were infected with high titers of the bacterium.

Statistical analyses (Fisher-Freeman-Halton test followed by adjusted residual analyses) on data pooled from the two trees (Fig. 3C) showed no significant deviation from expected infection patterns across the three symptom categories (Cat. A, B, and C; BH-adjusted *P* > 0.05). Only in asymptomatic leaves (Asymp.) was the proportion of samples carrying high *C*Las titers significantly lower than expected (BH-adjusted *P* < 0.05). Asymptomatic leaves were also the only group in which *C*Las-negative samples were observed (Fig. 3).

### Prophage typing of *C*Las detected in leaves showing different symptom types

**Fig 4:**
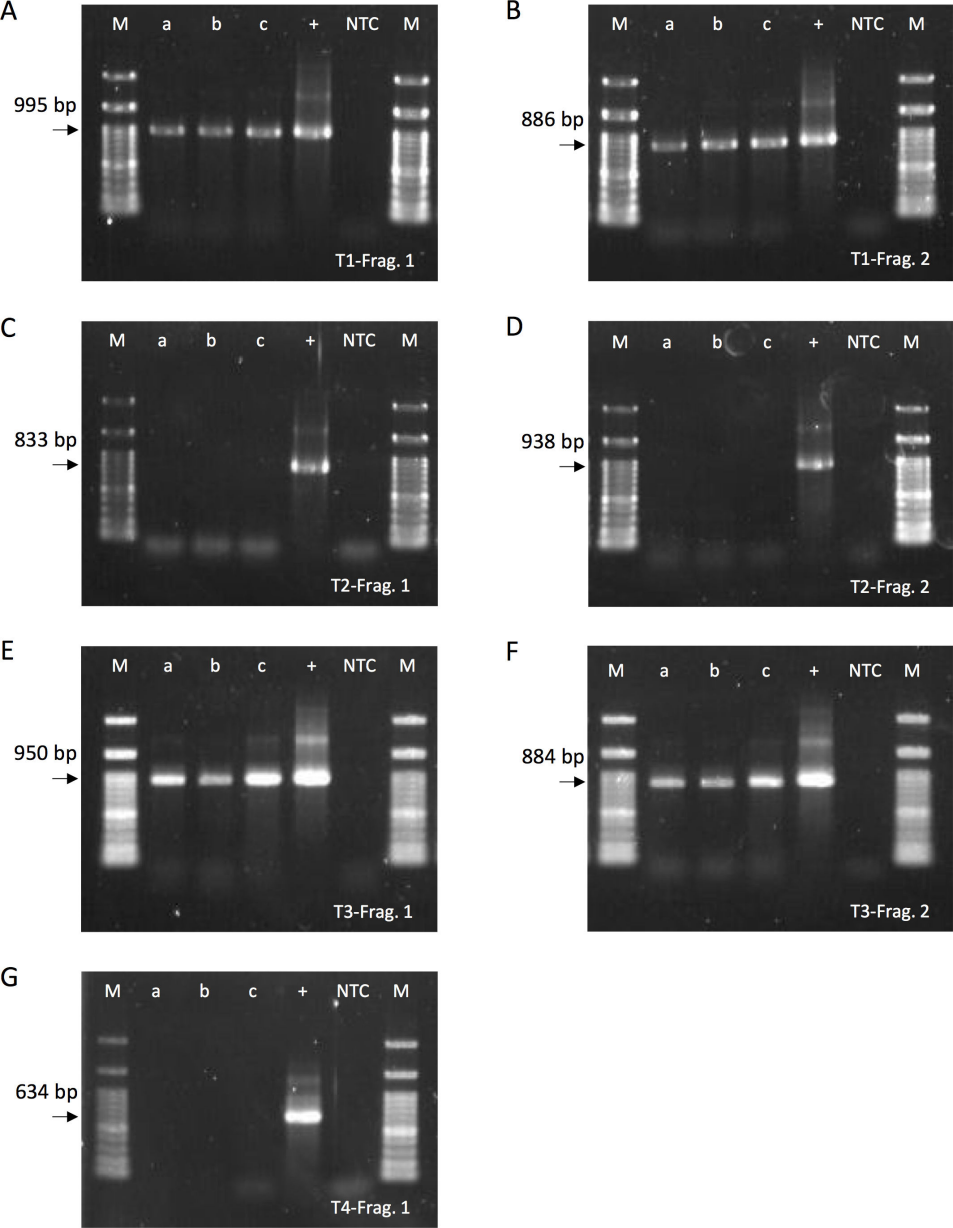
Detection of genetic fragments of Type 1 to Type 4 prophages in *C*Las-positive samples collected in January 2024. Prophage typing of *C*Las-positive DNA samples (Lanes a through c) were carried out using primers specific to fragments of (**A and B**) Type 1, (**C and D**) Type 2, (**E and F**) Type 3, and (**G**) Type 4 prophages of *C*Las. For these assays, gBlocks DNA fragments containing each targeted amplicon served as positive controls.

To determine whether differences in leaf symptoms were associated with the types of prophages present in *C*Las, leaf samples collected in January 2024 that were tested *C*Las-positive with PCR were subjected to prophage typing analyses targeting Type 1 to Type 4 prophages. PCR tests using type-specific primers were conducted, and all tested samples (15 Cat. A, 11 Cat. B, and 11 Cat. C samples) were found to contain both Type 1 and Type 3 prophages (Fig. 4), but not the other two prophage types. In one specific Cat. C sample, only one of the two Type 3 prophage genetic target, T3-Frag. 2, successfully amplified; PCR targeting the other fragment failed to produce any amplicon (Fig. S3). In the remaining samples, all Type 1 and Type 3 prophage fragments targeted in this experiment produced the expected amplicons (Fig. 4).

### Leaf nutrient analyses

**Fig 5:**
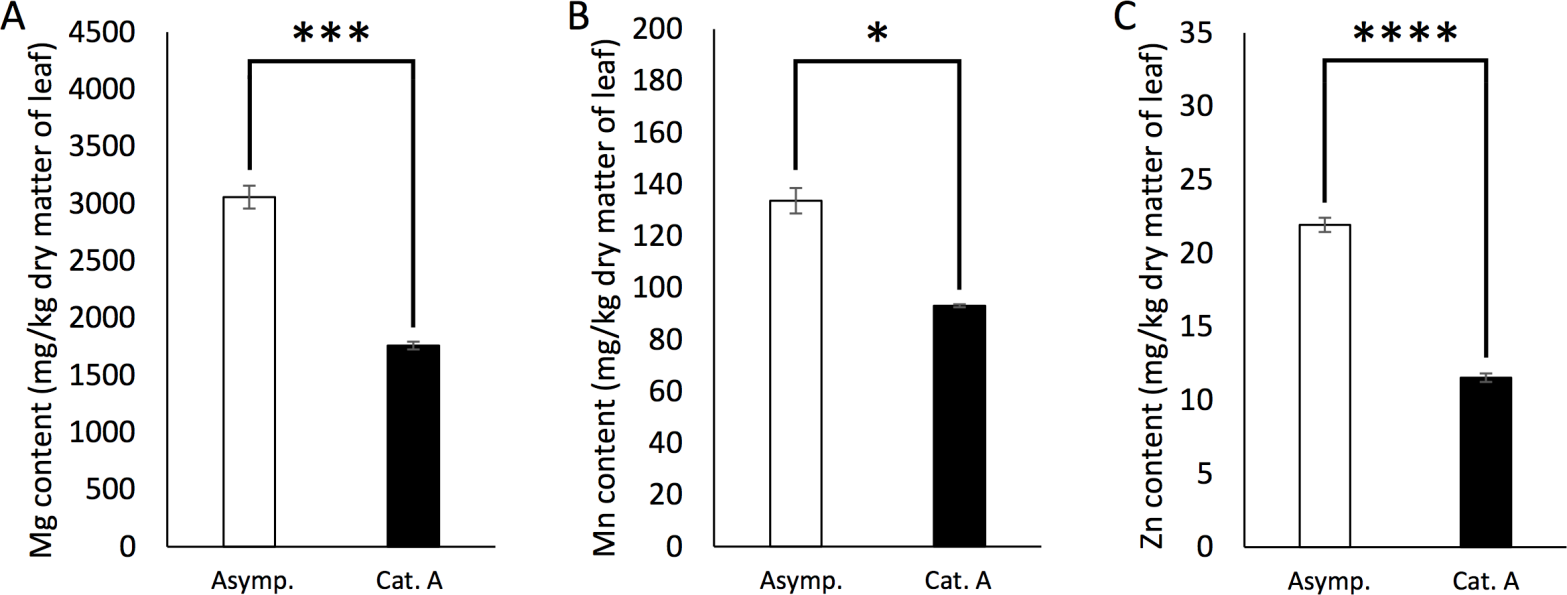
Results of plant nutrient analyses showing the (**A**) magnesium (Mg), (**B**) manganese (Mn), and (**C**) zinc (Zn) contents in Ponkan mandarin leaves that were either asymptomatic (Asymp.) or exhibited interveinal chlorosis (Cat. A). Differences between the two groups were determined with either *t*-tests (magnesium and zinc) or a Welch’s *t*-test (manganese), depending on the characteristics of the data. *P < 0.05, **P < 0.01, ***P < 0.001, ****P < 0.0001.

Because interveinal chlorosis has often been associated with deficiencies in nutrients, such as zinc, magnesium, and manganese (46–48), leaf nutrient analyses were conducted on Cat. A leaves (showing interveinal chlorosis) and asymptomatic leaves. Compared with asymptomatic leaves, Cat. A leaves had significantly lower levels of magnesium (*P* = 0.0003; *t*-test), manganese (*P* = 0.013; Welch’s *t*-test), and zinc contents (*P* < 0.0001; *t*-test; Fig. 5).

## DISCUSSION

Analyses of 16S rRNA gene sequences revealed that the Ponkan mandarin grove investigated in this study was affected by a strain of *C*Las; this result was consistent with previous reports on the distribution of *C*Las and the absence of *C*Lam and *C*Laf in Taiwan (4–6, 49, 50). The sequence of the detected strain was also identical to multiple published *C*Las strains, supporting current views on the low genetic diversity of *C*Las worldwide (24, 51).

Grove-wide PCR analyses of *C*Las detection rates in Ponkan mandarin indicated that in both warmer (August 2023; 27.8°C) and cooler (January 2024; 12.9°C) seasons, leaves with interveinal chlorosis (Cat. A leaves; Fig. 1B and C) were more likely to carry *C*Las than the other two symptom categories. Further grove-level qPCR analyses not only revealed consistent trends in *C*Las detection rates but also showed that Cat. A leaves had greater chances of detecting high *C*Las titers (Fig. 2). These patterns differed from those in a study on the *C. sinensis* cultivar “Hongjiang” (17), where the highest *C*Las detection rates were found in blotchy mottled leaves, which is widely recognized as the most typical leaf symptom of HLB (2, 4, 6, 35). These inconsistencies suggest that associations between leaf symptom expression and *C*Las infection patterns can differ depending on citrus cultivar or perhaps geographic region. Other interesting patterns also emerged when analyzing the obtained data at the canopy level. Samples from trees bearing Cat. A leaves were more prone to be tested *C*Las-positive and more likely to carrying high *C*Las loads, regardless of the diversity of symptoms found on the trees, indicating that interveinal chlorosis can be an indicator for *C*Las infection at different scales of observation in Ponkan mandarin.

Data from samples collected from the same trees exhibiting all three symptom categories revealed that Cat. A leaves still had the greatest likelihood of detecting high *C*Las titers when controlling for tree difference (Fig. 3). However, *C*Las detection rates and high-titer infection frequencies in all symptom categories were much greater than those found in their respective groups in the grove-wide analyses (Fig. 1B and C and Fig. 2A), which primarily surveyed trees with lower symptom diversity. *C*Las infection among asymptomatic leaves was also common in this sample set (Fig. 3), while in the grove-wide data set, almost all asymptomatic leaves from symptomless trees were *C*Las-negative. These trends were consistent with grove-level PCR and qPCR results showing that *C*Las detection rates and high-titer infection rates in leaves of all symptom categories were greater when they were collected from trees exhibiting more than one type of leaf chlorosis (Fig. 1E through G and Fig. 2B through D). Leaf symptom expression in HLB-affected citrus has been found to change as the disease progress (35), and in some cultivars, *C*Las titers can continue to increase post-infection (52, 53). Based on the correlations found in this study, high leaf symptom heterogeneity in the canopy may serve as an indicator for more severe or later-stage HLB infections. This possibility should be taken into consideration when evaluating HLB progression in field-grown Ponkan mandarin.

Prophage type difference in *C*Las has been shown to influence leaf symptom expression within the “Nianju” cultivar of *C. reticulata* (31). In this study, symptom variation in Ponkan mandarin was independent of prophage type difference (Fig. 4), as strains from different symptom categories all belonged to PTG1-3. The specific PTG has previously been detected in areas including India, China, and California (23, 25, 27), and perhaps further investigation may help to determine whether PTG1-3 strains from different regions are related to one another. Among the strains identified in the tested grove, there was one that only amplified one of the two Type 3 prophage fragments targeted (Fig. S3). Genetic variation within the same prophage type has been reported in earlier works and was suggested to be associated with genetic recombination or rearrangement events (54). Our data showed that in Ponkan mandarin, PTG1-3 *C*Las strains can exhibit such genetic heterogeneity within the same grove, but at a low frequency.

Interveinal chlorosis in citrus is often associated with deficiencies of nutrients, including magnesium, manganese, and zinc (46, 48). Comparison of nutrient contents between asymptomatic leaves and leaves with interveinal chlorosis (Cat. A) symptoms showed that the contents of all three nutrients were significantly lower in Cat. A leaves (Fig. 5), which had the highest *C*Las detection rates. Studies have shown that in addition to disrupting phloem transportation of photosynthates, HLB often led to deficiencies of nutrients, including magnesium, manganese, and zinc (47, 55). Some studies suggested that decline in the root system after *C*Las infection may negatively impact nutrient uptake (55, 56). It is therefore possible that the higher frequency of *C*Las infection (and high-titer infection) in Cat. A leaves contributed to the reduced nutrient contents detected in this category. There is also the possibility that nutrient deficiency can alter the plants’ susceptibility to *C*Las infection (55). Although it is unclear which of these explanations apply to Ponkan mandarin, the obtained results indicate that in groves growing this cultivar, leaves showing interveinal chlorosis should not be dismissed as merely signs of abiotic stress.

It is well known that *C*Las is primarily vectored by *D. citri* (12). During our four sampling sessions, however, no psyllid adults or nymphs were observed on the trees surveyed. The absence of psyllid vectors may have an effect on the *C*Las distribution patterns detected in this grove because oviposition and feeding of psyllids have been found associated with the *in planta* distribution and titers of liberibacters (57, 58). Specifically, oviposition of female psyllids and feeding of nymphs mostly occur on flushes and younger tissues, and the detection rates and likelihood of high-titer infection of liberibacters were found to be greater in these plant parts (57, 58). As such, we cannot rule out the possibility that different patterns could be observed if psyllid vectors were present. Additional studies on Ponkan mandarin groves affected by both *C*Las and their psyllid vectors can help to verify if this is the case.

Citrus production systems can vary greatly among different parts of the world (59, 60). Some areas cultivate citrus in large-scale groves on flat terrain (60), whereas others (such as the case in Taiwan) may rely heavily on smaller hillside production systems (59). These differences may influence local environmental conditions, cultural practices, and perhaps disease symptom expression. The grove examined in this study was in a hillside at approximately 481–535 m above sea level, which is substantially different from the low-elevation, large-scale citrus production systems commonly found in other citrus-growing regions such as Florida (61). These differences may contribute to variation in drainage, wind exposure, and potentially citrus physiology. Additionally, the fine-textured colluvial soils of the studied grove differ from the soil types commonly associated with many other citrus production areas (60, 61). Although growers in different citrus-producing areas routinely fertilize citrus trees (including those managing the studied grove), soil type differences may influence soil nutrient retention and the effects of fertilizers (62). These factors should be considered when interpreting or applying the findings from this study.

Finally, some previous observations have suggested that certain HLB leaf symptom types may appear sequentially during disease development (35). Slight variation may also exist within seemingly similar symptom categories (based on our observation). Therefore, it is possible that some of the patterns found among symptom categories in this study partly reflected differences in disease development stage rather than completely independent symptom types and may also have been influenced by variation within symptom categories. While the order of symptom appearance could not be definitively confirmed for the studied grove, attempts were made to maintain classification consistency by having all samples grouped by a single individual. In addition, two independent sample sets collected in 2023 and 2024 showed consistent trends, supporting the reproducibility of the patterns. Therefore, the associations found in this study should at least be applicable to Ponkan mandarin under similar field conditions for diagnostic purposes.

In conclusion, this study demonstrated that *C*Las infection status in Ponkan mandarin is correlated with symptom presentation at both the leaf and the canopy level. Symptom variation was independent of prophage type difference, but nutrient deficiencies may be involved in symptom expression. Since the patterns found in Ponkan mandarin was different from those reported in other citrus cultivars (17), further investigation on more citrus crops in distinct geographic regions could be needed for understanding the diversity of *C*Las infection dynamics under field conditions. The findings from this and future studies may be useful for symptom interpretation or developing symptom-based sampling strategies for *C*Las detection.

## MATERIALS AND METHODS

### Study site

The Ponkan mandarin grove studied in this work was located in Zhuolan Township, Miaoli County (120°51′58″E, 24°18′29″N; 0.7 ha in size), on a south-facing hillside at about 481–535 m above sea level. The average annual rainfall during 2023–2024 was around 1,931 mm, based on data from a weather station in Zhuolan Township (Climate observation data inquire service; https://codis.cwa.gov.tw/StationData). The grove had moderately fine-textured colluvial soil (pH = 4.1–4.5; according to the Taiwan Soil Survey Geographic Database; https://tssurgo.tari.gov.tw/Tssurgo/Map). Around 145–150 trees were grown in this grove, with slight yearly variation due to tree loss or replanting. According to the growers, most trees were more than 5 years old.

In terms of cultural practices, organic fertilizer was applied once per year. Micronutrients were sprayed monthly during the floral differentiation stage (December to the following January), and three to four times during the fruit enlargement stage (June to July). Compound fertilizers (containing nitrogen, phosphorus, and potassium) were applied twice before June each year, and after that, mostly calcium- and potassium-based fertilizers were used. Annual grove sanitation was conducted in early February, during which branches and trees with severe HLB-like symptoms were removed. Starting in 2023, dimethoate was applied to control *D. citri*, although, to the best of the growers’ knowledge, no psyllids have ever been found in the grove during the five years up to 2024.

### Sampling of Ponkan mandarin leaves

Four sampling sessions were conducted from August 2021 to September 2024 (Table 1) in the grove of interest. During each sampling session, the leaves were collected within the same day. All leaf samples were hand-collected. Details on each batch of the leaves sampled are described in the following sections.

**TABLE 1:** Information on the Ponkan mandarin (*Citrus reticulata* cv. Ponkan) leaf samples collected in this study

Time of sampling	Number of leaves sampled^*a*^	Usage
Aug 2021	65	Assessing the prevalence of HLB infection in the grove and identifying the liberibacter associated with disease symptoms
Aug 2023	163	Investigating liberibacter infection patterns in the grove and their association with leaf symptom variation (using PCR)
Jan 2024	182	Investigating liberibacter infection patterns in the grove and their association with leaf symptom variation (using PCR and qPCR)
Sep 2024	80	Investigating liberibacter infection patterns in leaves of different symptom categories within the same trees
	600	Leaf nutrient analyses

^*a*^ For leaves sampled for PCR and qPCR assays, a single leaf was used to extract one DNA sample; for leaves collected for leaf nutrient analyses, 100 leaves were pooled into one sample.

### Confirming the presence and identity of the liberibacter affecting the Ponkan mandarin grove

In August 2021, a total of 65 unfolded leaves were randomly sampled from different Ponkan mandarin trees (one leaf per tree). For symptomatic trees, leaves showing chlorotic symptoms, without detailed categorization, were collected. In terms of symptomless trees, asymptomatic leaves were collected randomly. For each leaf, midrib and leaf blade tissues were collected and stored at −80°C. DNA was extracted from the samples using a Synergy Plant DNA Extraction Kit (OPS Diagnostics, Lebanon, NJ). Extracted DNA samples were quantified with a Nanodrop One Spectrophotometer (Thermo Fisher Scientific, Waltham, MA). Undiluted leaf DNA samples (on average 108.2 ng/μL) were subjected to PCR testing using primers LG774F and LG1463R, which are specific to liberibacters’ 16S rRNA gene (genus-specific; Table 2). DNA samples of liberibacter-infected Ponkan mandarin leaves initially provided by the grower served as positive controls. The expected amplicon size for this assay was around 684 bp. Representative liberibacter-positive samples were subjected to sequencing. The sequences obtained (639 bp after removing primer-binding sites) were searched against GenBank using BLASTN.

**TABLE 2:** Primers and probes used in this study

Organism-target gene/genetic fragment	Primer/probe name: sequence (5′ to 3′)	Final conc.	Expected product size (bp)	PCR condition	Source
‘*Ca*. Liberibacter’—16S rRNA gene	LG774F: GTAAACGATGAGTGCTAGCTGTTGGG LG1463R: CTGACCRTACCGTGGCCGG	0.25 µM	684	1 × 95°C 3 min, 35 × (95°C 30 s, 62°C 30 s, 72°C 1 min), 1 × 72°C 5 min	(45)
*Citrus reticulata*—resistance protein-like protein gene	P-B5_2F: CAGCAGAGTCATGGAAGGATAAA P-B5_2R: CCATTGAACCGCAAACATTAAGA	0.4 µM	204	1 × 95°C 3 min, 30 × (95°C 30 s, 56°C 30 s, 72°C 20s), 1 × 72°C 5 min	This study.
*Ca*. Liberibacter asiaticus (*C*Las)—outer membrane protein gene	omp F: GTGATTCTGAGGGTGAGCG omp R: CGAACTCACTGAGAACTGATC	0.4 µM	693	1 × 95°C 2 min, 35 × (95°C 40 s, 55°C 30 s, 72°C 1 min), 1 × 72°C 10 min	(63)
*C*Las—16S rRNA gene	HLBas_c: TCGAGCGCGTATGCGAATAC HLBr: GCGTTATCCCGTAGAAAAAGGTAG HLBp: AGACGGGTGAGTAACGCG	0.1 µM	76	1 × 50°C 2 min, 1 × 95°C 10 min, 40 × (95°C 15 s, 60°C 1 min)	(64)
*C*. *reticulata—LEAFY* gene	LFY_q1F: AGGCCGGAGCGAGCTATATT LFY_q1R: CATGCCCCAACATTCTCCCC	0.1 µM	136	1 × 95°C 3 min, 40 × (95°C 10 s, 58°C 30s)	This study.
*C*Las type 1 prophage—T1-Frag. 1	SC1-2F: TGGCTCGGGTTCAGGTAAAT SC1-2R: AAGGGCGACGCATGTATTTC	0.2 µM	995	1 × 95°C 5 min, 35 × (95°C 30 s, 60°C 30 s, 72°C 1 min), 1 × 72°C 10 min	(65)
*C*Las Type 1 prophage—T1-Frag. 2	SC1-3F: CTCACTGCGTCTTGATTCGG SC1-3R: CGAACGAGCGGTATGTTTGT	0.2 µM	886	1 × 95°C 5 min, 35 × (95°C 30 s, 60°C 30 s, 72°C 1 min), 1 × 72°C 10 min	(65)
*C*Las Type 2 prophage—T2-Frag. 1	SC2-2F: ACCCTCGCACCATCATGTTA SC2-2R: TCGTCTTGATTGGGCAGAGT	0.2 µM	833	1 × 95°C 5 min, 35 × (95°C 30 s, 60°C 30 s, 72°C 1 min), 1 × 72°C 10 min	(65)
*C*Las Type 2 prophage—T2-Frag. 2	SC2-3F: ACAGTTAAGAGCCACGGTGA SC2-3R: AAGACGTGGGTGTTATGGGT	0.2 µM	938	1 × 95°C 5 min, 35 × (95°C 30 s, 60°C 30 s, 72°C 1 min), 1 × 72°C 10 min	(65)
*C*Las Type 3 prophage—T3-Frag. 1	891-1F: CTGATCCTTTACCATGCCGC 891-1R: CAGCGAAACCGATCTTGAGG	0.2 µM	950	1 × 95°C 5 min, 35 × (95°C 30 s, 60°C 30 s, 72°C 1 min), 1 × 72°C 10 min	(29)
*C*Las Type 3 prophage—T3-Frag. 2	891-2F: ACCGCGATCTACCCGTAATT 891-2R: TGTGTTTTGCGAGTGAAGGG	0.2 µM	884	1 × 95°C 5 min, 35 × (95°C 30 s, 60°C 30 s, 72°C 1 min), 1 × 72°C 10 min	(29)
*C*Las Type 4 prophage—T4-Frag. 1	T4P2F: GTGAAGGAGACCAAAGTGAGAA T4P2R: CGACCACATGACCAGACTTATT	0.2 µM	634	1 × 95°C 5 min, 35 × (95°C 30 s, 60°C 30 s, 72°C 40 s), 1 × 72°C 10 min	This study.

### Determining *C*Las infection rates in Ponkan mandarin leaves of different symptom categories

#### Collection of leaf samples

Associations between leaf symptom variation and *C*Las infection patterns were determined using leaf samples collected in August 2023 and January 2024. The two timepoints were selected to determine whether the associations remained consistent across different temperature conditions. The average temperatures in August 2023 and January 2024 were around 27.8°C and 12.9°C, respectively.

Symptomatic leaves were collected so that one leaf belonging to each symptom category available on each tree was sampled (avoiding pseudoreplication due to sampling multiple leaves from the same tree). Asymptomatic leaves were collected from trees without observable chlorotic symptoms (one leaf per tree). Samples collected for this experiment were all unfolded leaves. In total, 163 and 182 leaves were sampled in August 2023 and January 2024 from 143 and 148 trees, respectively. Among the collected leaves, 75 and 137, respectively sampled from 55 and 103 symptomatic trees in August 2023 and January 2024, were symptomatic (chlorotic). Further leaf symptom categorization into Cat. A, Cat. B, and Cat. C was conducted by a single individual throughout the study to maintain consistent classification standards.

#### Determining CLas infection rates with PCR

DNA was extracted from the entire midrib of each leaf with the Synergy Plant DNA Extraction Kit. The samples for these assays were quantified and diluted to the same concentration (20 ng/μL). Initial conventional PCR tests targeting a Ponkan mandarin gene were conducted to ensure that the qualities of the DNA samples were sufficient for PCR assays; primers P-B5_2F and P-B5_2R, designed from the sequence of a resistance protein-like protein gene (GenBank accession no. KJ019188.1; Table 2), were used in these tests. PCR with primer pair omp F/omp R targeting the outer membrane protein gene (*omp*) of *C*Las (Table 2) was used to determine the pathogen’s infection rates. The expected amplicon was around 693 bp in size. The assays were conducted with the GoTaq Green Master Mix (Promega, Madison, WI) in 25-μL reactions. Each reaction included 20 ng of template DNA. The PCR conditions for each type of assay are shown in Table 2. A *C*Las-positive DNA sample from the first sampling session (August 2021) was included as a positive control. The amplification results were examined with gel electrophoresis. The *C*Las detection rates were analyzed using chi-square tests and, when necessary, pairwise chi-square tests with BH adjustments. The detection rates in leaves of different symptom categories obtained from different “tree types” were compared using Fisher-Freeman-Halton tests and, when needed, pairwise Fisher’s exact tests with BH adjustments.

### Quantification of *C*Las titers in leaves of different symptom categories

qPCR tests coupled with plasmid-based standard curves were conducted on DNA extracted from samples collected in January 2024. This sample set was subjected to qPCR testing because it had more balanced proportions across different leaf symptom categories, and the overall sample size was larger. For each sample, the copy numbers of *C*Las’s 16S rRNA gene (if detected) and Ponkan mandarin’s *LEAFY* gene were determined. *C*Las gene copy numbers were determined with TaqMan assays using primer pair HLBas_c/HLBr and the probe HLBp, and Ponkan mandarin’s *LEAFY* gene copy numbers were quantified with SYBR green tests using primers LFY_q1F and LFY_q1R (Table 2). The TaqMan and SYBR green tests were respectively conducted with the iTaq Universal Probes Supermix (Bio-Rad, Hercules, CA) and the iQ SYBR Green Supermix; both using a CFX Connect Real-Time PCR Detection System (Bio-Rad, Hercules, CA). The thermocycling programs for these tests are shown in Table 2. All assays were carried out in 20-μL reactions, including 20 ng of template DNA. Absolute quantifications of both target genes were achieved by cloning the target amplicons into *Escherichia coli* JM109 using the pGEM-T Easy Vector System (Promega, Madison, WI) and linearizing extracted plasmids using PstI-HF (New England Biolabs, Ipswich, MA). Linearized plasmids were recovered, quantified, diluted, and used to construct standard curves in every run, as previously described (66). Aliquots of the same preparation of linearized plasmid were used in the same experiment to ensure the comparability among data from different qPCR runs. In each type of assay, three technical replicates were included for every sample and linearized plasmid standard. Data from the qPCR tests were used to calculate gene copy numbers. *C*Las population densities were determined by dividing *C*Las’s 16S rRNA gene copy number by that of the Ponkan mandarin *LEAFY* gene for each sample (i.e., expressed as a standardized pathogen-to-host gene copy number ratio). The data from this study were obtained from qPCR runs with efficiencies above 85% (R^2^ > 0.999). Based on the data, three infection statuses were designated, these included undetected (zero value), low-titer infection (population densities >0 and <1), and high-titer (population densities ≥1). The data were analyzed using a Fisher-Freeman-Halton test and adjusted residual analyses with BH adjustments.

### Comparison of *C*Las titers in leaves of different symptom categories within the same trees

In September 2024, two plants, Tree no. 1 and Tree no. 2, bearing leaves of all three symptom categories (i.e., two plants belonging to “Tree type ABC”) were subjected to additional sampling. Ten leaves were collected for every leaf symptom category from each tree. Ten asymptomatic leaves were also collected from each of both trees. The samples were subjected to DNA extraction and qPCR testing using methods described above. The data were analyzed using a Fisher-Freeman-Halton test and adjusted residual analyses with BH adjustments.

### Prophage typing of *C*Las

For prophage typing, conventional PCR assays targeting two genetic fragments specific to each of the Type 1, 2, and 3 prophages were conducted using primers designed in a previous study (29, 65). For Type 4 prophages, a specific primer pair was designed based on sequence data available in GenBank (accession no. CP045566; Table 2). Details on the prophage genetic fragments targeted in this study are shown in Table S1. Conventional PCR assays were conducted with the GoTaq Green Master Mix. The final concentrations of the forward and reverse primers in each reaction (25 μL in volume) were 0.2 μM, and the amount of template DNA added to each reaction was 20 ng. For these assays, synthetic gBlocks DNA fragments containing the targeted gene fragments (Integrated DNA Technologies, Coralville, IA; Table S1) were used as positive controls (20 ng per reaction). The thermal cycling conditions of these assays are shown in Table 2. All PCR products were analyzed with gel electrophoresis.

### Leaf nutrient analyses

To determine whether nutrient deficiencies could be detected in Cat. A leaves, we examined whether zinc, magnesium, and manganese contents were significantly different between asymptomatic and Cat. A leaves. Due to limitations on leaf collection during the production season, nutrient analyses were not extended to Cat. B and Cat. C leaves.

Briefly, 300 asymptomatic leaves and 300 leaves showing Cat. A symptom was randomly collected from the targeted grove. Leaves belonging to each group were divided into three replicates, each including 100 leaves. Leaf nutrient analyses were conducted by the Soil Survey and Testing Center at National Chung Hsing University, Taiwan. Collected leaf samples were oven‐dried 70°C and ground to fine powder. The samples were then passed through a 35‐mesh sieve to ensure homogeneity. For each sample, 0.4 g of the powder was wet-digested in HNO₃:HClO₄ (5:1 v/v) at 180°C until completely digested. The resulting samples were then diluted and inductively coupled plasma with atomic emission spectroscopy (ICP‐AES) was used to determine the magnesium, manganese (Ultima 2; HORIBA, Minami-Ku, Kyoto, Japan), and zinc (Ultima Expert; HORIBA, Minami-Ku, Kyoto, Japan) contents. Data from the nutrient analyses were analyzed using independent sample *t*-tests or Welch’s *t*-test.

## Data Availability

The sequence obtained in this study has been deposited in GenBank under the accession number PX363072.

## SUPPLEMENTAL MATERIAL

Supplemental figures and table spectrum.00760-26-s0001.docx Figures S1 to S3 and Table S1.

ASM does not own the copyrights to Supplemental Material that may be linked to, or accessed through, an article. The authors have granted ASM a non-exclusive, world-wide license to publish the Supplemental Material files. Please contact the corresponding author directly for reuse.
