# Supplementary material for: Leaf- and canopy-level chlorotic symptom variations are associated with differential Candidatus Liberibacter asiaticus infection patterns in Ponkan mandarin under field conditions
Source: Microbiol Spectr. 2026 Jul 2;14(8):e00760-26. doi: 10.1128/spectrum.00760-26 (PMC13436140; doi:10.1128/spectrum.00760-26)
Supplement: Supplementary file 1 [file spectrum.00760-26-s0001.docx]

**Supplementary materials**

**Fig. S1**. Results of PCR assays targeting liberibacter’s 16S rRNA gene. The assays were conducted on Ponkan mandarin leaf DNA samples collected in August 2021. Only results of representative samples (Lanes 1 through 14) are shown. For these tests, DNA samples of *C*Las-positive Ponkan mandarin leaves collected before the study (July 2021) served as positive controls (+). M: 100-bp DNA marker; NTC: no-template control.

**Fig. S2**. (A) Samples containing *C*Las’s DNA amplified an expected amplicon around 693 bp in size. Only results of representative samples are shown (Lanes 1 through 10). (B) Results of PCR assays targeting Ponkan mandarin’s resistance protein-like protein gene. The tests amplified an expected 204-bp amplicon in all tested Ponkan mandarin leaf DNA samples. +: a liberibacter-positive Ponkan mandarin leaf DNA sample collected in August 2021; M: 100-bp DNA marker; NTC: no-template control.

**Fig. S3**. Detection of Type 3 prophage’s genetic fragments revealed that (A) one specific *C*Las-positive sample collected in 2024 (Lane 3) failed to amplify T3-Frag. 1. (B) However, T3-Frag. 2 successfully amplified in this sample. For these assays, gBlocks DNA fragments containing each targeted amplicon served as positive controls (+). M: 100-bp DNA marker; NTC: no-template control.

**Table S1**. Sequences of the gBlocks DNA fragments used in the prophage typing assays.

| Prophage type | Target genetic fragment (GenBank accession no.) | Sequence (5’ to 3’)^1^ |
| --- | --- | --- |
| Type 1 | T1-Frag. 1 (NC_019549) | AGGGGCTCACTTGGTTTTTGTCTATCGGAATGCTCTTTGTACTGCGGTAG**TGGCTCGGGTTCAGGTAAATGAGGTTTAAAATGAGGATCTTCAAGGGTGTTGTCGGCTATGGTTTGAAGCTTTTCTTGATCAAAATGTGGATACTCACCCCTAACCAAGCTATCAACCCCATGAGCTAAAGTATCCGTATGAGCCTCATAAGCATCAAAGCTGGTATGCAAGCCAGGGCTAGAAGATTTAACCCCATGTTTATAGGGTAATCGCTCCGTAATACCCTCTTTCAAATCGTTAACAAGACGCAAAGACATATTTTGTACTTGCTTTGAATGCATTCCGCCGAAGAATGCACCGATCAGACCATCTGTTATCAGAGATTCCATATCAAAGATGCGGTAATGCTGTGCCATATCCGGATATCCATGATCTTCTAAAACTTTGGACGACCAACCCCGTTCAACCATACCAAAAGGAACATTCAACACTGCCCCACTTGCGACGGTCTTCGCTATGGATTGAGAAGCAATAGCGCCAGGAGCGAGTAACGCAGACGTATGCACTATCGCCTCCCGCCACGCTAAAGCATCAGCGGTCTCTTTATCCACTCCTTCTATTTGATGGTGTATTGAGCTCTCAGCTTTATGGGATAAGTAAGCATATAATGCTCCTGCTGCTAGTGGAGCGGATTGCAAAGCAAGCCCCGCTAATCGGGTCAGAGGTGTTGGTATAAAGCTTAGCAACTTCCCCGCAAGAGCCGCCCCCGCAATATAAGGAGCAAGGCTTGTTAATCCTTCAACAAGATGCGCCCCAGTCCCTACAGAATGAGGATCCGTACGGGAACCCCTGTAATAATTGGGTTGGTCGTGCGTCTCTTCCCGAAAGGGAGCAACAAGCTTATCTAAAGACCTTGCGGGCATGTTTATAACTTCTTTACCAAGCCCCGTGTGCCATTTGATATCAGGGGAAACACGTGGTCTTTGCGCCCATTCCTTAATATTATCTCTTATATCCTCATCGCTTACCGCGTTGAAATACATGCGTCGCCCTT**TCTGTTTTACGGTGCGTGTTCGTTAAAAATTTGGTGTTCATAGTGCCTTA |
|  | T1-Frag. 2  (NC_019549) | CAAGAGCGTCCCCGAGATCACGCAACCCCGCTTGAGGATTGACGGAGTCC**CTCACTGCGTCTTGATTCGGAGTAACCGCCTCCACTAAGGGTTTGTTGGTCGTGATCTCAATCATGAAGCGTCCTTTCCTTTTACGTTATAACGTCTAAACATCTTCATACCCGCCGATGACGCCGACTCTACCATAGGAGGACCGGCGATTGCGGCAACGGACGCCCATCTATTATTTTTAACCGCTTCCTTATTAGCTCTGTGCCATCCTGCTTCTTTAGCAAACCGAGCAACCGTTTGTTCTCTCGCTGTTCGAGCGGTGTTGATACCTTTATAGGCGTTGCGGGTGTTTTGCCCGACCAACAGATCCAAACTCGCCCCCGATACACCCGATAACACCGCTTTCATACGAAAGACACCCGTGTCCATTATCCCTTCTCTGCGGGCTTGGTCTTCCCTATCCAAATAGAGAACATCCGCCCTAAGAGCGTTTTCTTCCGCTAGTAATGATCGATATTCGTGGTCTCTAATCGACGACCTGTGCGAAGCAAGCGTCGAAGAAATACGAAGCCCAGCTCCAACAAGGGGTGACGCAAGTGATAATCCATATAAGAGGTCATCCATGCCTATGCTCATATCGCTCTCCTTCAAATTGCGTGTGTGCGGTGATGGAAGTAAGACAGAAATAACCTCCGGTCTTTTGGCGGATGATTATTTCAGGGTTAAGCGACGCATCGGACGGGACTAAAACCTCAAATTCGCCCGTCTTCGGTTTGCCTCGTAACTCCTCCACAGGGACAATAGGAAAACCTAATATCCCAACTTCAAGATCAGCGGTGTTGATAACTCGAACCACTACCTTGAATATACGACGCTTCTTACCCATTGTGCTGTTGAAGGACTCACTCGTATCCATACTGGGAATACAAACATACCGCTCGTTCG**CACCCCCTTCATATTCATCCTCTTTTGATCCATCATCTTTTGCCAACCTT |
| Type 2 | T2-Frag. 1 (NC_019550) | TTTAAAATTGCGAGTAAAACCACCGACATAATCTTGTAAGGTGGCAGATA**ACCCTCGCACCATCATGTTATCTAATGTGGTCAACCCCTGCCAATCCATGGTCTTTTTCGCCATAACCCCGAGAACAGACTGACTATTTGTTTCTAAATCACGGGTAAAACCCGTCGCCATATGTTCTAAACCAACTGTTATACTTTTTATAAATTCTTTTCTCTCTTTGCCTGCCACTGGGGAAGCATTCCAAATATTCGCAATATTCGTTTTCCACGTATATCCCATCCGTTGAGAACCCATAAAAGCACTCTCAATAATTTCTTTAGGTATATACAACGATTGGCTACCCAACTTAGTTATAACCGTCCAAGCTGTCGCAGAATCAACAATTTTATCCATGGTAGAAGGTTGTTTTCTTCCCATTGTCAATCCATCCCAATCCATTTGGACTTGCATCTCTTCCTTTTTCAGCTTATTAAGAGCTTTTGTTTTTTTATCCCCTTCTAAACGACTGTTAATATCTTCTCGATCTCTCTCAAACATACTTGCTAAAACAAAGTTAAAATTCTCATTTGCATTAGCCCCAAACTCACGAGCTATGGCAATATCCCTTATGAGATTATCAAACGAACGACTCATCATTCCTTCAACAGTCAAATCAGAACCAAACTCTTTCATCGCCTCAACCAAACCTTGAGGGGTGTAGTGAAGTTGACGAACTTGACGTAATCGACCTCCAAGAGAAATCCCAGAATGATCCTTCCCTCCTGATGCAATAACCTTGTTACGACCCTCACTTGCTAATGTCTCATAAACATCTTCAACAAATTGACGCAATGATCCTTCATTATCCAGTCCTCTTTTTTGATACTCTGCCCAATCAAGACGA**GGAAGGGTGCGATCAACGAATTCATCTTTCGTCACTAACGATATCTTCCG |
|  | T2-Frag. 2  (NC_019550) | TACACGAACTGCATTAATACATTCCTCTTTCATCCTTTCACTCCAAATAA**ACAGTTAAGAGCCACGGTGAATACATCCTTTTTAGACAATTCAATATCTCTGGTAATATGCTCTTTAATTCCTGGTTCATGAGCAAATTGCTGTTCTAAATCGGTCAATCTACGTTCGTATTCAGGAACTTGAGAAGATAAAGGGTCTTGTTCCTTTAAAGCTTTCGGTTTTGAAACCTCCTGCTGAGCAAGGAAATCCTCCCCTTGTTTTAACTGTTTCTTCAACTCAGGACTGAACTCTACTTCAGGTTTGGTAATCATATCCTTGGTCATTAAATCTATCGACTTTGGATCAACATTAATTTCTTCGCCTCGCCTCATTTTCTCAATGGCTTGTTCTAATGTCTGAGCATGAAGTTCAGCCGATCTAGAGGTAGTCGCAATAGCAGGAGCAGAATGGTATATATCATCAATATCTCTTTTAACCACTTGAGCTATATCAACATCAGAAGGTTTGATATCCGGATGTTTCCCTCCTTTACCATGCAACGCTCCTAATCCCGCACCGATAATAAACTCCGTCGTTGTATGGATACCATCCACTTCCCTATAATGTTGGGCTAAATCCTTGTGACCTTTATAATCTAAATACGCACCTATGCTTTGTCGCTCCCCTAATCCAAAAGCAACATTTGTCGCCCCGCCAGTAACCGCCTTTGACACCAATGATTTACCAATAACACCAGAAACACTACCTCCTGCAAAAGTAGTTCCTGTCGTTATTAAAGCTCCTATCTTGGCAGTCTCATGCGCAACCCCCTCATCCCTTAATTCTTTATATCTTCTCGTCCCTGATGCGGATGATGCAGTCGCTAGTCCTATGAAGGGGGAAGCAGGAAGAGTAACTGGGTTAATAGCGAATAGACTAAAACCTGTCCCAAATGCTGATAAAAAATGACCAATGCCATGAGCGACACCACCCATAACACCCACGTCTT**CAGCAACATTATACCAAGGTTTACTGGTAATCTTATTATGAGGGGTATAA |
| Type 3 | T3-Frag. 1 (KY661963) | GACAGGAATTAAGAATTTCAAGATATCCTGCCTGCTTCCTACCGGAATGA**CTGATCCTTTACCATGCCGCACGTTATAGGCAAAAAAACCATCCGATGCCAAACAATAATACAGAAATTTCGATACGATTGCGTGTCGAAATTGATTTTTAATTCGAATAGCGAGAACATTTCCGCTGCATCCGCCGTTGTTATCAGCAAACCATATCTTTTTGAGACAAGGTCGCATATTGCCCAATAAAATATCTCCTCTTTTATAACTCGTTAATTTATTCACAGTTGGCACATAGCTTGAATCAATCTTACCTTTTGCATTTTGCAACAAATTATCAACACCAACAAAAGTTGTACAATCAAGTTCACTAGAATTAATACGTGTGTCAGAATAATATCCTACTTCTCCCAATTCTTTCCACTCTGCTTCCTGAGAAAAACTTGAAAACAATTGTTGCGTCAGCCCTTTTTTGGGTGCTTTAAGGGCGTTGTTCTTGTCACAATTTGCGCTCTCACATTCAGAGAACACGGTGTTGCTTAATGTTGAATACAAGAGTTCAGCGCCGGCAAGCACATCAGAAGAGATCAAATCCACCTTAGCCGCCGCTTTAAAAAGACGTGTCAATTCAATTTGTTTTTTCAAACAGGGGATCAAGAATTCTTGCTCGGCTAACACTTTCCACTTGATTGTCGGAGAAAGCGATCCCGATGATACAGATATTGCCCGATTCATAAAGACATCAGACTGCATAAAGAACGGCAAAAAATCAGAGATTACGTATTCGGGATTCGCTTTAAGGACCATAGCATGATGTGAGCACATGCAATCCCAATCGGATACGGCAACTTTGCGCAGATAAGCACGACGCTTGCAAAAAATAATCTGCCCTTTTTTTACCAATAACTTTTGCCCCTCAACATCGCTTGGTACACCGTGACGCTTAATCTTCAGGCTGTTTGTGTTTAAATGTTCTAAACCAATATAAACCTCAAGATCGGTTTCGCTG**GGCTTCACACGTTTCGAAATATGTTTTGCAATATCACCAAACTTTACCCT |
|  | T3-Frag. 2 (KY661963) | TTCATCAAGATTGTTTTCTTTGGCTAGTTTCCCAAACTCCTCGTTGATTGC**ACCGCGATCTACCCGTAATTCGGCTAGACGCACTTGAAACTTCACGGGATTGGTTGCACCGTCTCGGATAGATTGTTTATAGCTGTAGCGACTCATGTAACGGCCGACGTCTTTCTCGCTTCCAAACAAATGAAATGTATTACGATCCCATTTTGAAATCGGTGTACCGGTTAATCCGTAAAAATGAGCATTCGGCAAAGCCCAACGCATTTTCTCTCCCAAATCACCTTCTTGAGTTCGATGCGCTTCGTCCACCAATACAATGATATTATCGCGGTTATTGAGTCCATTGGGATTGCTCTCATCAATCTTAACATCTTTAAACTTAAAGACGGTTGTGATAAAAATTCCACGGTTATCCTTTGCAATACAATCACCAAGCTCTTTACAACTTTGCACTTGGATGAGATTCTTAACGTCGGCCCCGCCGAAAGTTTCGTTGATCTGGCTGTCTAAATCTCGGCGGTCTACTACCACCAATACCGTCGGATTCTTAAGTTGATTGTCGGAACGTAATATTTTAGCGGCATAGAGCATCAGTAAAGACTTACCGGATCCCTGAAAATGCCAAATCAGTCCTTTCTTTGTATCACCTTGACGAACCCTCTCAACAATCTGTTTCGCCGCTTCAAATTGCGGGTAGCGCGGTAACATTTTCATGAGTTTAGAAGTTTCCTTACCGGTTCTTCTCATTGAAAAAAGAGTAAATGATTTCAGAATCTGCAATAAAGTTTGCGGGCTTAATAAACTTTTACAGCTTTCCAAAACCGATTCCAAGCCGAAAACGTCACTTCGTAAGCTGGTATGATGCCAAGGTGCCCAATCTTTGAAACGGGTATGAACGGAACCGTAAGCAAAAGTTTTCCCTTCACTCGCAAAACACA**AAAGGTTCGGCACAAAAAAAGGCTCTGCATTACGCCAGTAGTGCTTTTTC |
| Type 4 | T4-Frag. 1 (CP045566) | TGCTTGCAACAGAATTTGCTCCTCATAATTTGTCTCGTCCGCCGAGACAT**GTGAAGGAGACCAAAGTGAGAAGTCGTTTTGGTAATTTTTTTAATCCTTTTACAATGCATAATCGTGCTTTTTATGGAAGATATCAGGAAATTGATGAAAATACTAAGCGGTTTTATAATGCTTTGCCGTGGCAGTACAGACGATGGAAGGATAAGAAAACAGGTGAGTGGCATAATAAAACCATTGATCCTATGGTTATGTGGAGGCAAGTGTTTAAACATGGTGATAAACTTGATTTTGTGAAGGTTTTGGAAAATGCCCTTTGGGATGAAGTGACTGATCGATATATAGGTTTTTTTGGTCGTGTTATGTCTAAGGTTTCTAGGAAGCTTGGAATTCCTCATTTTTTTGGTTTAGATAGTTTGTCTTATCAATATAAGAAGTTTGAAGTTGCTAAGAAGCTTGGTATAGAAGATAAATTTGGTTCTGATAGGCCATTGAGTACGGCTCATGTGGTTAATACTATTGATGATCTTTATATGCGTGTTTCTGGTTCTGAAAATGGTTTTTTGAAGGATTTTAAGGAAACTATTAAGCATTCTGATGCGTATAAGGAGTTGACTGATTATATGCGATCTCATTTGTATGAGCGTCATAAAGAAGATATTAAGAATAAGTCTGGTCATGTGGTCG**TTAAGAAAGGTGATTTTAAAGTTTCGAATCAGTACCTTTTTGATTATTTGAAGAGAACTAGTGTTGTTAAAAATATAAATGATTATTTCAATCCGCCTAAACCTGCGTATAAACCTATGTATGAGCATCCTCTAACTGTTCATGAAAGAGGTATTGCTGCTGATCGAGAGAGATATCGGAGGTTTCAGGCTAAGTTGGCGAGAGAGAAAAAGAAAAGGGATG |

^1^ The amplified fragments are indicated with bold font. The primer-binding sites are underlined.
